# Smart Energy Harvesting for Internet of Things Networks

**DOI:** 10.3390/s21082755

**Published:** 2021-04-13

**Authors:** Fisayo Sangoleye, Nafis Irtija, Eirini Eleni Tsiropoulou

**Affiliations:** Department of Electrical and Computer Engineering, University of New Mexico, Albuquerque, NM 87131, USA; fsangoleye@unm.edu (F.S.); nafis@unm.edu (N.I.)

**Keywords:** network economics, energy harvesting, wireless powered communications systems, contract theory, artificial intelligence, reinforcement learning, Internet of Things

## Abstract

In this article, we address the problem of prolonging the battery life of Internet of Things (IoT) nodes by introducing a smart energy harvesting framework for IoT networks supported by femtocell access points (FAPs) based on the principles of Contract Theory and Reinforcement Learning. Initially, the IoT nodes’ social and physical characteristics are identified and captured through the concept of IoT node types. Then, Contract Theory is adopted to capture the interactions among the FAPs, who provide personalized rewards, i.e., charging power, to the IoT nodes to incentivize them to invest their effort, i.e., transmission power, to report their data to the FAPs. The IoT nodes’ and FAPs’ contract-theoretic utility functions are formulated, following the network economic concept of the involved entities’ personalized profit. A contract-theoretic optimization problem is introduced to determine the optimal personalized contracts among each IoT node connected to a FAP, i.e., a pair of transmission and charging power, aiming to jointly guarantee the optimal satisfaction of all the involved entities in the examined IoT system. An artificial intelligent framework based on reinforcement learning is introduced to support the IoT nodes’ autonomous association to the most beneficial FAP in terms of long-term gained rewards. Finally, a detailed simulation and comparative results are presented to show the pure operation performance of the proposed framework, as well as its drawbacks and benefits, compared to other approaches. Our findings show that the personalized contracts offered to the IoT nodes outperform by a factor of four compared to an agnostic type approach in terms of the achieved IoT system’s social welfare.

## 1. Introduction

Internet of Things (IoT) has gained great research and industrial interest in the last decade, as it enables the operation and collaboration of a large number of devices with different communication and computing capabilities, such as sensors, actuators, smartphones, and others [1]. Those IoT devices collect and report information to several types of application in order to support the end-users’ needs and deliver meaningful services, such as environmental monitoring, social networking and surveillance systems [2]. The exploitation of the IoT devices’ physical and social characteristics can create efficient coalitions among them, to better serve a common goal in the system, e.g., crowdsourcing, surveillance of an area of interest, and in-home healthcare [3]. A common characteristic of the IoT devices is their frequent transmission of data to a receiver, e.g., access point, a multi-access edge computing server, for further processing and planning of the delivered services [4]. Even though the amount of transmitted data is usually small, the frequent transmissions reduce the battery life of the IoT devices, which often have limited power resources, and their battery replacement is a difficult and costly task [5]. Thus, the energy harvesting solution from radio frequency signals by deploying a wireless powered communication system has arisen as a suitable means to prolong the IoT devices’ battery life [6]. In this paper, we introduce a smart energy harvesting method by exploiting the principles of *Contact Theory*, and an artificial intelligent model to support the autonomous IoT nodes’ association to femtocell access points based on *Reinforcement Learning*.

### 1.1. Related Work

The topic of energy harvesting by IoT devices has been thoroughly studied in the literature, mainly focusing on the technical and implementation aspects of the problem [7]. The authors in [8] identify the problem of the limited battery of the IoT nodes and they provide a short survey regarding the existing energy harvesting technologies, and the corresponding power management techniques to sparingly use the harvested energy. In [9], the authors aim to jointly optimize the data flow from the IoT nodes to the users and the IoT nodes’ battery usage, by deploying IoT gateways and energy transmitters to save the energy used for the transmissions and charge the IoT nodes in parallel, respectively. Furthermore, a game-theoretic approach is adopted based on the theory of Stackelberg games, where the IoT gateways optimize the data caching and incentivize the energy transmitters to charge the IoT nodes, by determining their optimal transmission power strategy. A detailed survey study is presented in [10] that identifies the currently available IoT energy harvesting systems, the corresponding energy distribution approaches, and the energy storage devices and control units that facilitate the IoT nodes’ energy harvesting process. The provided categorization of the energy harvesting systems enables the reader to identify the differences among the existing energy harvesting techniques and the corresponding energy distribution approaches, concluding with the most appropriate selection per realistic use case scenario. A predictive energy harvesting model is introduced in [11] by exploiting the extended Kalman filtering method and jointly guaranteeing the Quality of Service (QoS) requirements and several security protection levels in the IoT system. The proposed predictive energy harvesting model can enable the IoT system to plan its energy harvesting needs per connected IoT node and proactively adapt its operation and energy consumption based on the trade-off of energy demand and energy availability.

The exploitation of multiple energy harvesting sources and techniques, such as solar, radio frequency, thermal, artificial light, is studied in [12], by introducing a hybrid energy harvesting model for the IoT nodes that can jointly support the energy harvesting from several sources of energy. The authors provide a mathematical analysis to prove the energy harvesting benefits in terms of the amount of the harvested energy and the efficiency in the energy harvesting process via multiple and hybrid energy harvesting sources compared to a single source of energy harvesting. Focusing on wireless powered communications systems and radio frequency energy harvesting, the authors, in [13], describe a game-theoretic and labor economics-based approach to deal with the optimal energy harvesting under complete and incomplete information scenarios, respectively, regarding the channel conditions among the IoT nodes and the energy transmitters. A Lyapunov optimization-based approach is formulated in [14] to jointly optimize the frequency and the stability of the sampling rate of the IoT energy harvesting nodes showing the increase in the amount of harvested energy. The main novelty of the proposed method is its real time operation and adaptation to the IoT system’s conditions and energy availability of the nodes and the access points without making any assumptions nor predictions on future energy availability patterns. Furthermore, an on-demand energy harvesting model is proposed in [15] towards improving the delay performance of the radio frequency energy harvesting process by introducing two associated discrete time Markov chain models that jointly optimize the average packet delay, the packet loss probability, and the network throughput. The novel concept of directed radio frequency signals charging in a unicast manner each IoT node is also introduced in [15]. The proposed method can charge each IoT node in a personalized manner by transmitting directed radio-frequency beams to the node, thus, increase the amount of harvested energy by the IoT node.

A deep reinforcement learning approach of the actor-critic deep Q-network reinforcement learning algorithms [16] is presented in [17] to jointly address the access and power transmission and harvesting problem of the IoT nodes by considering the sum rate and prediction loss. The importance of IoT energy harvesting nodes in public safety scenarios is discussed in [18], where the IoT nodes create coalitions among each other based on their physical and socio-technical characteristics, which are further exploited by a mobile Unmanned Aerial Vehicle (UAV) in order to select the IoT node cluster that will be charged. This research work is extended in [19] by jointly optimizing the nodes’ transmission power to further report their data to an access point. Additionally, an energy-harvesting-aware routing algorithm is presented in [20] to jointly improve the IoT nodes’ battery life and the IoT network’s Quality of Service under different traffic loads and energy availability conditions. A practical application on IoT energy harvesting nodes is introduced in [21], where the IoT nodes measure the vibration conditions of railway tracks, and report them to a reader, in order to monitor the railway track conditions. The IoT nodes are installed on the railway tracks and harvest radio frequency energy from a reader installed on the train.

Following the above analysis, it is concluded that great attention has been devoted to the technical and implementation aspects of energy harvesting in IoT systems. Specifically, the most recent approaches that have been reviewed above mainly focus on the improvement in the amount of harvested energy, either by providing directed radio-frequency beams from the transmitter to the receiver, or by improving the efficiency of the allocated charging power to the IoT nodes, or even by optimizing the energy consumption of the IoT nodes; thus, greater energy availability is achieved. However, the reviewed approaches have not fully exploited the IoT nodes’ physical and social characteristics during the energy harvesting process, and their interactions with the energy transmitters [22], in order to ultimately optimize the enrgy harvesting process.

To address these issues, in this paper, we design a contract-theoretic approach to capture the interactions among the IoT energy harvesting nodes and the energy transmitters [23,24]. Our goal is to determine the optimal IoT nodes’ harvested energy with respect to the amount of data that they transmit, and the energy transmitters’ optimal charging power. We also introduce an artificial-intelligence-based mechanism to enable the IoT devices to select the most beneficial energy transmitter based on their energy harvesting experience [25].

### 1.2. Contributions & Outline

The increasing number of Internet of Things (IoT) nodes and their corresponding need to extend their battery life in order to support IoT services have highlighted, which has elevated the need to address the problem of energy harvesting from radio frequency signals in a wireless powered communication system. The ultimate goal of this approach is to guarantee the smooth operation of the overall IoT system and prolong its seamless operation. To the best of our knowledge, this is the first research work that systematically studies the energy harvesting process in an IoT system from a techno-economics and artificial intelligent point of view. We introduce the concept of IoT energy harvesting node types, which are expressed as a function of their communication interest, proximity to the energy transmitter and each other, and their energy conversion efficiency. The IoT nodes’ and the access points’ utility functions are designed to represent the profit of the different entities from the energy harvesting and data acquisition process, respectively. The main contributions of this paper are summarized as follows:

1. Based on the principles of Contract Theory, an optimization problem is formulated and solved to determine the IoT nodes’ transmission power, transmitted data to the associated access point, and the energy transmitters’ optimal charging power, in order for the overall system to converge to an optimal and stable point of operation;
2. An artificial-intelligence-based reinforcement learning mechanism is introduced, which targets the most beneficial long-term energy transmitter selection from each IoT energy harvesting node in an autonomous and distributed manner.

The rest of this paper is organized as follows. The system model is discussed in Section 2. The IoT node types and all the involved entities’ utility functions are presented in Section 3.1. The contract-theoretic optimization problem is formulated in Section 3.2 and solved in Section 3.3. The artificial intelligent energy transmitters’ selection by the IoT nodes is discussed in Section 4. Numerical results are presented in Section 5, and the conclusions are drawn in Section 6.

## 2. System Model

**Figure 1:**
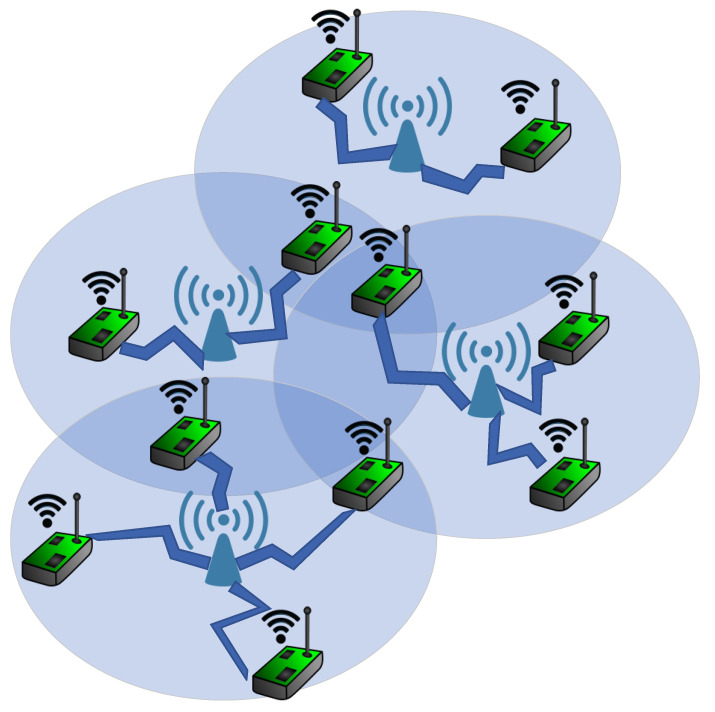
Smart Energy Harvesting for Internet of Things Networks.

A femtocell-based communications network is considered consisting of |F| femtocells with overlapping coverage range in the examined communications environment and their set is F={1,…,f,…,|F|}. The femtocell access points (FAPs) jointly act as data receivers from the connected IoT nodes and energy transmitters [26]. A set of IoT energy harvesting nodes I={1,…,i,…,|I|} is considered. The distance among two IoT nodes $i,i^{\prime }\in I$ is denoted as $d_{i,i^{\prime }}\left[m\right]$, while the distance of an IoT node from a FAP is $d_{i,f}\left[m\right],\forall i\in I,\forall f\in F$. The overall system operates as a wireless powered communication network (WPCN), where the Wireless Energy Transfer (WET), and the Wireless Information Transmission (WIT) phases are executed within a timeslot τ[sec]. The WET and WIT phases’ duration is denoted as $\tau_{WET}\left[sec\right]$ and $\tau_{WIT}\left[sec\right]$, respectively, with $\tau =\tau_{WET}+\tau_{WIT}$. The considered system model is presented in Figure 1.

The IoT nodes can communicate among each other in order to exchange the information needed to perform a task, e.g., temperature sensors measuring the temperature in a smart building [27]. We define the relationship factor $r_{i,i^{\prime }}\in \left[0,1\right]$ among two IoT nodes. A higher value of the relationship factor shows a higher level of communication interest among two IoT nodes. The communication channel gain conditions among two IoT nodes and among an IoT node and a FAP are defined as $G_{i,i^{\prime }}=\frac{\lambda }{d_{i,i^{\prime }}^{2}}$, $G_{i,f}=\frac{\mu }{d_{i,f}^{2}}$, respectively, where λ,μ>0 capture the fading phenomena. At each timeslot τ, each IoT node has some available energy $E_{av.i}^{\left(\tau \right)}\left[J\right]$, which indicates its maximum possible transmission power during the WIT phase, as $P_{i}^{Max(\tau )}=E_{av.i}^{\left(\tau \right)}\cdot \tau_{WIT}\left[W\right]$. Each IoT node harvests $E_{harv.i}^{\left(\tau \right)}\left[J\right]$ energy during the WET phase, and invests $E_{tr.i}^{\left(\tau \right)}\left[J\right]$ energy to transmit its data to the FAP during the WIT phase. Thus, the available energy of each IoT node for the next timeslot τ+1, is determined as $E_{av.i}^{\left(\tau +1\right)}=E_{av.i}^{\left(\tau \right)}+E_{harv.i}^{\left(\tau \right)}-E_{tr.i}^{\left(\tau \right)}$. The transmission power of the IoT node *i*, in order to report its data to the FAP *f*, is denoted as $P_{i,f}\left[W\right]$, while the personalized FAP’s charging power for the IoT node *i* is $P_{f,i}\left[W\right]$. The FAP uses directional beams in order to improve the efficiency of the energy’s harvesting [15]. Considering the non-orthogonal multiple access (NOMA) technique in the uplink communication from the IoT nodes to the FAPs, and the Successive Interference Cancellation (SIC) technique implemented at the FAPS, each IoT node’s achievable data rate is given as follows based on Shannon’s formula [28]

(1) $$\;R_{i,f}=W\cdot \log\left(1+\frac{P_{i,f}\cdot G_{i,f}}{\sum_{i^{\prime }\geq i+1}P_{i^{\prime },f}\cdot G_{i^{\prime },f}+\sigma^{2}}\right)\;$$

where *W* [Hz] is the system’s bandwidth and $\sigma^{2}$ is the power of zero-mean Additive White Gaussian Noise (AWGN). It is noted that without loss of generality, we consider $G_{|I|,f}\leq \cdots \leq G_{i,f}\leq \cdots \leq G_{1,f}$, thus, by implementing the SIC technique, the signal of the IoT node with the highest channel gain is decoded first at the corresponding FAP, as presented in Equation (1). Given that the IoT devices reside in a small area, we account for the interference stemming from all the IoT nodes’ transmissions, even if they are connected in different FAPs [29]. The acronyms and the notation adopted in this paper are presented in Table 1 and Table 2, respectively.

## 3. Contract Theoretic Energy Harvesting

In this section, we will exploit the principles of Contract Theory towards capturing the interactions among the IoT energy harvesting nodes and the FAPs, in terms of transmitting data and harvesting energy and charging the nodes, respectively. Assuming that each IoT node has selected the FAP that it will communicate with and harvest energy from (details in Section 4), each FAP acts as a virtual “employer”, offering personalized rewards to each connected IoT node, in terms of charging power towards incentivizing the nodes, which act as virtual “employees”, to invest an effort—translated in their transmission power—to report their collected data to the FAP for further exploitation by the IoT service that is offered to the end-users, e.g., smart heating systems.

### 3.1. Types, Utility Functions, and Contracts

Each IoT node is characterized by its type, which depends on the node’s physical and social characteristics within the IoT network. Those characteristics are summarized in the socio-physical factor $SP_{i}$, the proximity factor $\rho_{i,f}$, and the energy conversion efficiency factor $\eta_{i}$. Towards building the socio-physical factor $SP_{i}$ for each node *i*, we initially consider the channel gain symmetric matrix $G={\left\{G_{i,i^{\prime }}\right\}}_{\left|I|\times |I\right|},\forall i,i^{\prime }\in I$, and create the channel quality vector $G_{\Sigma }=\left[\sum_{i=1}^{\left|I\right|}G_{1,i},\ldots ,\sum_{i=1}^{\left|I\right|}G_{|I|,i}\right]$. The latter is a simple and indicative factor of the communication channel conditions of each node *i* with all the other IoT nodes within the examined IoT network. We normalize the channel quality vector, as ${\tilde{G}}_{\Sigma }=\left[{\tilde{G}}_{1},\ldots ,{\tilde{G}}_{\left|I\right|}\right]$, where ${\tilde{G}}_{i}=\frac{G_{\Sigma }^{\left(i\right)}}{\sum_{i=1}^{\left|I\right|}G_{\Sigma }^{\left(i\right)}}\in \left[0,1\right]$ and $G_{\Sigma }^{\left(i\right)}=\sum_{j=1}^{\left|I\right|}G_{i,j}$. Furthermore, we consider the communication interest factor $CI_{i,i^{\prime }}\in \left[0,1\right]$ among two IoT nodes $i,i^{\prime },\forall i,i^{\prime }\in I$, capturing the need of two IoT nodes to exchange information among each other in order to perform an IoT service. We define the communication interest symmetric matrix $CI={\left\{CI_{i,i^{\prime }}\right\}}_{\left|I|\times |I\right|},\forall i,i^{\prime }\in I$ and create the communication interest vector $\mathrm{CI}=[\sum_{i=1}^{\left|I\right|}CI_{1,i},\ldots ,\sum_{i=1}^{\left|I\right|}CI_{|I|,i}]$. Thus, we obtain the normalized communication interest vector $\tilde{\mathrm{CI}}=\left[{\tilde{CI}}_{1},\ldots ,{\tilde{CI}}_{\left|I\right|}\right]$, where ${\tilde{CI}}_{i}=\frac{\sum_{i^{\prime }=1}^{\left|I\right|}CI_{i,i^{\prime }}}{\sum_{i=1}^{\left|I\right|}\sum_{i^{\prime }=1}^{\left|I\right|}CI_{i,i^{\prime }}}\in \left[0,1\right]$ shows the relative communication interest of each node *i* with all the other IoT nodes in the network. By jointly combining the normalized communication interest and channel quality indicators, we conclude with the socio-physical factor $SP_{i}={\tilde{G}}_{i}\cdot {\tilde{CI}}_{i},SP_{i}\in \left[0,1\right]$.

Additionally, each node *i* being associated with FAP *f* is characterized by the proximity factor $\rho_{i,f}\in \left[0,1\right]$, which expresses the node *i*’s normalized distance from the FAP *f*, with respect to the FAP’s maximum coverage range. Each node is characterized by its energy conversion efficiency factor $\eta_{i}\in \left[0,1\right]$, which shows how efficiently the node can convert the harvested energy from the FAP’s directed radio frequency beam to energy that can be exploited for its operations, e.g., data transmission. Considering the aforementioned three factors, the type of each IoT node is defined as follows

(2) $$t_{i}=SP_{i}\cdot \rho_{i,f}\cdot \eta_{i}$$

Each node invests an effort $q_{i}\in \left[0,1\right]$ in order to transmit its data to the FAP, which is translated to its uplink transmission power $P_{i,f}=q_{i}\cdot P_{i}^{Max}$. For simplicity in the notation, we have omitted the timeslot τ indicator in the rest of the analysis. Furthermore, the FAP incentivizes each IoT node, which is connected to this FAP, to report its data by charging it with directed radio frequency beams. The FAP’s personalized reward to the node *i* is denoted as $r_{i}\in \left[0,1\right]$, and the corresponding power of the directed radio frequency beam is $P_{f,i}=r_{i}\cdot P_{f}$, where $P_{f}\left[W\right]$ is the FAP *f*’s available charging power. Thus, the IoT node’s harvested energy in a timeslot τ during the WET phase, as discussed in Section 2, is $E_{harv.i}^{\left(\tau \right)}=\eta_{i}P_{f,i}G_{i,f}\cdot \tau_{WET}$, while the corresponding energy invested to its data transmission during the WIT phase is $E_{tr.i}^{\left(\tau \right)}=P_{i,f}\cdot \tau_{WIT}$.

Each IoT node evaluates the received reward $r_{i}$ from the FAP based on the evaluation function on $e(r_{i}\left(t_{i}\right))$, which is a strictly increasing function with respect to the received reward, e.g., $e\left(r_{i}\left(t_{i}\right)\right)=\sqrt{r_{i}\left(t_{i}\right)}$. In practice, the evaluation function captures the node’s required charging power. Therefore, each IoT node’s utility function is defined by the revenue that the IoT node enjoys from the charging process (first term of Equation (3)), while considering the cost of its data transmission due to its invested transmission power (second term of Equation (3))

(3) $$\;U_{i}\left(t_{i},r_{i},q_{i}\right)=t_{i}e\left(r_{i}\left(t_{i}\right)\right)-kq_{i}\left(t_{i}\right)\;$$

where $k\in R^{+}$ is the IoT node’s experienced cost to transmit its data by investing its transmission power.

Focusing on the benefit of each FAP from collecting data from the IoT nodes, we express its utility as the profit gained from the IoT nodes’ invested effort, while considering the cost to provide the rewards. Each FAP is not aware of the IoT nodes’ type; thus, we define the probability $Pr_{i}\left(t_{i}\right)$, with $\sum_{i=1}^{\left|I\right|}Pr_{i}\left(t_{i}\right)=1$, that node *i* is of type $t_{i}$. Therefore, each FAP’s f,∀f∈F, utility function is defined as follows

(4) $$\;U_{f}\left(t,r,q\right)=\sum_{i=1}^{\left|I\right|}\left[Pr_{i}\left(t_{i}\right)\left(q_{i}\left(t_{i}\right)-wr_{i}\left(t_{i}\right)\right)\right]\;$$

where $t=\left[t_{1},\ldots ,t_{\left|I\right|}\right],r=\left[r_{1},\ldots ,r_{\left|I\right|}\right],q=\left[q_{1},\ldots ,q_{\left|I\right|}\right]$ are the IoT node types, rewards and effort vectors, respectively, and $w\in R^{+}$ is the FAP’s cost of providing the rewards, due to the spending energy required to perform the node charging.

### 3.2. Problem Formulation

In this section, we will formulate the problem of optimal energy harvesting and charging as a contract-theoretic optimization problem, as follows.

(5a) $$\max_{\begin{array}{c} \left\{r_{i},q_{i}\right\}\\ \forall i\in I \end{array}}U_{f}\left(t,r,q\right)=\sum_{i=1}^{\left|I\right|}\left[Pr_{i}\left(t_{i}\right)\left(q_{i}\left(t_{i}\right)-wr_{i}\left(t_{i}\right)\right)\right]$$

(5b) $$s.t.\;t_{i}e\left(r_{i}\right)-kq_{i}\geq 0,\;\forall i\in I$$

(5c) $$t_{i}e\left(r_{i}\right)-kq_{i}\left(t_{i}\right)\geq t_{i}e\left(r_{i^{\prime }}\right)-kq_{i^{\prime }},\;\;\forall i,i^{\prime }\in I,\;i\neq i^{\prime }$$

(5d) $$0\leq r_{1}<\ldots <r_{i}<\ldots <r_{\left|I\right|}$$

The solution to the optimization problem (5a)–(5d) is the optimal contract $\left\{r_{i}^{*},q_{i}^{*}\right\}$ for each IoT node i∈I.

In the following description, we discuss the physical meaning of thed optimization problem formulated above in detail. To determine the optimal harvested power by the IoT nodes, and the optimal charging power provided by each FAP to each connected IoT node, the profit/benefits of the FAPs and the IoT nodes should be jointly optimized, as presented in (5a)–(5d). Each FAP aims to optimize its utility function (5a) towards determining the optimal contract $\left\{r_{i}^{*},q_{i}^{*}\right\}$.

It should be noted that the optimization problem (5a)–(5d) is solved by each FAP and the corresponding IoT nodes connected to it. Thus, we solve as many optimization problems as the number of FAPs in the examined system, while considering that each IoT node should at least receive a positive utility (Equation (5b)) in order to be incentivized to participate in the IoT network. The latter condition (Equation (5b)) is referred as *Individual Rationality (IR)*. Furthermore, each node achieves a higher utility when receiving the contract designed for its unique characteristics, i.e., type, as compared to any other contract designed for another node (Equation (5c)). This condition is referred to as *Incentive Compatibility (IC)*.

Additionally, for notation convenience, we sort the types of the IoT nodes as $t_{1}<\ldots <t_{i}<\ldots <t_{\left|I\right|}$. Towards further elaborating on the constraint of Equation (5d), we analyze and prove the conditions of fairness, monotonicity, and rationality in the following three propositions.

**Proposition** **1.** *(Fairness) An IoT node of higher (or the same) type will receive a higher (or the same) reward, i.e., $r_{i}>r_{i^{\prime }}\Leftrightarrow t_{i}>t_{i^{\prime }}\;\;\left(r_{i}=r_{i^{\prime }}\Leftrightarrow t_{i}=t_{i^{\prime }}\right)$.*

**Proof.**  See Appendix A.1. □

Based on the fairness condition, an IoT node of a higher type, i.e., improved socio-physical characteristics, will enjoy higher reward from the FAP, i.e., increased charging power.

**Proposition** **2.** *(Monotonicity) An IoT node of higher type, i.e., $t_{1}<\ldots <t_{i}<\ldots <t_{\left|I\right|}$, will invest a higher effort, i.e., $q_{1}<\ldots <q_{i}<\ldots <q_{\left|I\right|}$.*

**Proof.**  See Appendix A.2. □

The physical meaning of the monotonicity property is that an IoT node of better socio-physical characteristics, i.e., type $t_{i}$, is expected to report greater amount of information by investing more uplink transmission power, i.e., effort $q_{i}$. Thus, the FAP will provide a greater reward $r_{i}$ by an increased charging power. The last condition that is examined is the rationality.

**Proposition** **3.** *(Rationality) An IoT node of higher type, i.e., $t_{1}<\ldots <t_{i}<\ldots <t_{\left|I\right|}$, will eventually experience higher utility, i.e., $U_{1}<\ldots <U_{i}<\ldots <U_{\left|I\right|}$.*

**Proof.**  See Appendix A.3. □

The conditions of fairness, monotonicity, and rationality are presented in a combined manner in Equation (5d).

### 3.3. Problem Solution

In this section, our goal is to solve the contract-theoretic optimization problem, as presented in Equations (5a)–(5d), under the scenarios of complete and incomplete information from the FAPs perspective regarding the IoT nodes’ socio-physical characteristics, i.e., types. The solution of the contract-theoretic optimization problems, which are solved by each FAP along with its connected IoT nodes, will result in determining the optimal contracts $\{r_{i}^{*},q_{i}^{*}\},\forall i\in I$. Based on this solution, the optimal charging power $P_{f,i}$ of each FAP to each connected node will be determined, as well as the optimal transmission power $P_{i,f}$ of each IoT node.

**Complete Information Scenario**: In this scenario, the FAPs know the types of the IoT nodes in a deterministic manner, thus, the contract-theoretic optimization problem (5a)–(5d) can be rewritten, as follows.

(6a) $$\max_{\begin{array}{c} \left\{r_{i},q_{i}\right\}\\ \forall i\in I \end{array}}\left[q_{i}-wr_{i}\left(q_{i}\right)\right]$$

(6b) $$s.t.\;t_{i}e\left(r_{i}\right)-kq_{i}\geq 0$$

**Theorem** **1.** ***(Optimal Contract under Complete Information)*** The optimal contract $\left\{r_{i}^{*},q_{i}^{*}\right\}$ among an IoT node i connected to the FAP f considering complete information of the IoT nodes’ types is $\left\{{\left(\frac{t_{i}}{2wk}\right)}^{2},\frac{t_{i}^{2}}{2wk^{2}}\right\}$.

**Proof.**  See Appendix A.4. □

The complete information scenario is an ideal case, and will mainly be used for benchmarking purposes. In practice, the FAPs have limited information regarding the IoT nodes’ socio-physical characteristics, i.e., types. Thus, in the following analysis, we examine the scenario of incomplete information regarding the IoT nodes’ types.

**Incomplete Information Scenario**: In the following analysis, we examine the contract-theoretic optimization problem that was presented in (5a)–(5d) under the incomplete information scenario. Initially, we perform a reduction in the individual rationality conditions in Equation (5b). Based on the monotonocity and incentive compatibility conditions, we have that: $t_{i}e\left(q_{i}\right)-kq_{i}\geq t_{i}e\left(q_{i^{\prime }}\right)-kq_{i^{\prime }}\geq t_{i}e\left(q_{1}\right)-kq_{1}$. Given that $t_{i}>t_{1}$, we can rewrite the above inequality as follows: $t_{i}e\left(q_{i}\right)-kq_{i}\geq t_{i}e\left(q_{1}\right)-kq_{1}\geq t_{1}e\left(q_{1}\right)-kq_{1}\geq 0$. Thus, we conclude that the individual rationality condition holds true for all the IoT nodes, if $t_{1}e\left(q_{1}\right)-kq_{1}\geq 0$ holds true. The latter constraint can be further reduced to $t_{1}e\left(q_{1}\right)-kq_{1}=0$, as the FAP will provide the minimum sufficient reward to the IoT nodes to participate in the IoT network. Thus, the constraint (5b) is equivalent to $t_{1}e\left(q_{1}\right)-kq_{1}=0$.

Next, our goal is to reduce the incentive compatibility (IC) constraints, as presented in Equation (5c). The following terminology is used in order to represent the IC constraints: (i) $i,i^{\prime },i^{\prime }\in \left\{1,\ldots ,i-1\right\}$: downward IC constraints; (ii) i,i−1,i∈I: local down IC constraints; (iii) $i,i^{\prime },i^{\prime }\in \left\{i+1,\ldots ,|I|\right\}$: upward IC constraints; and (iv) i,i+1,i∈I: local upward IC constraints.

**Lemma** **1.** *All the downward IC constraints are equivalent to the local downward IC constraint.*

**Proof.**  See Appendix A.5. □

Following the same philosophy, we state the following Lemma.

**Lemma** **2.** *All the upward IC constraints are equivalent to the local downward IC constraint.*

**Proof.**  See Appendix A.6. □

Based on the above analysis of reducing the constraints, we can rewrite the initial contract-theoretic optimization problem as follows:

(7a) $$\max_{\begin{array}{c} \left\{r_{i},q_{i}\right\}\\ \forall i\in I \end{array}}U_{f}\left(t,r,q\right)=\sum_{i=1}^{\left|I\right|}\left[Pr_{i}\left(t_{i}\right)\left(q_{i}\left(t_{i}\right)-wr_{i}\left(t_{i}\right)\right)\right]$$

(7b) $$s.t.\;t_{1}e\left(q_{1}\right)-kq_{1}=0,$$

(7c) $$t_{i}e\left(q_{i}\right)-kq_{i}=t_{i}e\left(q_{i-1}\right)-kq_{i-1}$$

(7d) $$0\leq r_{1}<\cdots <r_{i}<\cdots <r_{\left|I\right|}$$

We observe that the optimization problem (7a)–(7d) is a convex optimization problem. Therefore, to determine the optimal contracts $\{r_{i}^{*},q_{i}^{*}\},\forall i\in I$, we can use standard convex optimization techniques [30].

## 4. Artificial Intelligent Association

In this section, we introduce an artificial-intelligence-based reinforcement learning mechanism to enable the IoT nodes to make the most beneficial long-term energy transmitter (i.e., FAP) selection in an autonomous and distributed manner. Our study focuses on the Log-Linear reinforcement learning algorithms, such as the Max Log-Linear and the Binary Log-Linear algorithms, which are able to converge to the best equilibrium point (if one exists) of the system with high probability. Additionally, the Log-Linear algorithms allow the IoT nodes to deviate from their probabilistically optimal decisions and make some suboptimal decisions in order to thoroughly explore their available actions. In this paper, we adopt the Max Log-Linear mechanism that requires no exchange of information among the IoT nodes and the FAPs. Each IoT node aims to learn, in the long-term, the most-beneficial choice of FAP; thus, its strategy space is $S_{i}=\left\{s_{1},s_{2},\ldots ,s_{f},\ldots ,s_{\left|F\right|}\right\}$. Initially, each IoT node selects a strategy $s_{i}\in S_{i}$ with equal probability $P_{i}^{\left(ite=0\right)}\left(s_{i}^{\left(ite=0\right)}\right)=\frac{1}{|S_{i}|}$, where ite presents the iteration of the Max Log-Linear algorithm. Then, at each iteration, one IoT node is randomly selected to explore an alternative strategy ${s_{i}^{\prime }}^{\left(ite\right)}$ with equal probability $\frac{1}{|S_{i}|}$, and receives a corresponding utility ${U_{i}^{\prime }}^{\left(ite\right)}$. The selected IoT node updates its strategy following the probabilistic learning rules in Equation (8a) and Equation (8b), while the rest of the IoT nodes keep their previously selected strategies unchanged, i.e., learning phase.

(8a) $$P_{i}^{\left(ite\right)}\left(s_{i}^{\left(ite\right)}={s_{i}^{\prime }}^{\left(ite\right)}\right)=\frac{e^{\beta \cdot {U_{i}^{\prime }}^{\left(ite\right)}}}{\max\{e^{\beta \cdot U_{i}^{\left(ite-1\right)}},e^{\beta \cdot {U_{i}^{\prime }}^{\left(ite\right)}}\}}$$

(8b) $$P_{i}^{\left(ite\right)}\left(s_{i}^{\left(ite\right)}=s_{i}^{\left(ite-1\right)}\right)=\frac{e^{\beta \cdot U_{i}^{\left(ite-1\right)}}}{\max\{e^{\beta \cdot U_{i}^{\left(ite-1\right)}},e^{\beta \cdot {U_{i}^{\prime }}^{\left(ite\right)}}\}}$$

The pseudo-code of the introduced Max Log-Linear algorithm that enables the IoT nodes to select a FAP, which they can harvest energy from and communicate with the selected FAP, is presented in Algorithm 1. The outcome of the Max Log-Linear algorithm will be the stable selection of FAPs from the IoT nodes.
**Algorithm 1:** Max Log-Linear Algorithm
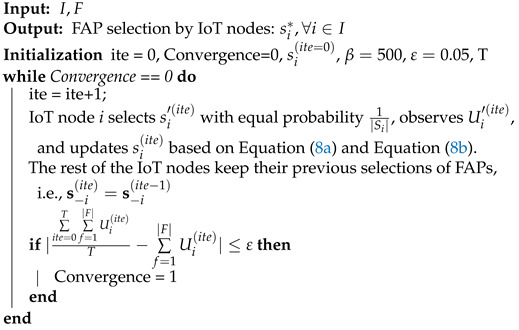



## 5. Numerical Results

In this section, a detailed numerical evaluation analysis is presented based on simulations in order to show the effectiveness and performance of the proposed smart energy harvesting framework for Internet of Things networks. First, in Section 5.1, we focus on validating the operation of the proposed contract-theoretic energy-harvesting mechanism, in terms of determining the optimal contracts under the scenarios of complete and incomplete information regarding the IoT nodes’ socio-physical characteristics. The benefits of adopting Contract Theory and exploiting the IoT nodes’ characteristics are presented in Section 5.2. Having verified and analyzed the pure operation of the proposed framework, a detailed comparative evaluation is presented in Section 5.3 to show the superior performance of the overall system by enabling the IoT nodes with artificial intelligence, against other approaches that have been used in the literature.

Throughout our evaluation, we consider |F|=5, |I|=100, τ=1 s, $\tau_{WIT}=0.3\tau$, $\tau_{WET}=0.7\tau$, $d_{i,i^{\prime }}\in \left[0,50\right]$ m, $d_{i,f}\in \left[0,50\right]$ m, λ=1, μ=1, η∈[0,1], $W=5\cdot 10^{6}$ Hz, $\sigma^{2}=10^{-15}$, $E_{av,i}^{\left(t=0\right)}\in \left[0,20\right]mJoule$ representing a typical IoT system consisting of IoT nodes, such as temperature sensors [31]. The proposed framework’s evaluation was conducted in an ACER laptop, with Intel Core i7, 3.9GHz Processor, and 16GB available RAM. In the following results, unless otherwise explicitly stated, the above values of the simulation parameters are used.

### 5.1. Pure Operation Performance

In this section, we present the pure operation performance of the proposed contract-theoretic energy harvesting model by examining the scenarios of complete and incomplete information of the IoT nodes’ characterises from the FAPs’ perspective. The results presented below are derived from one indicative timeslot, where the overall framework was executed, i.e., IoT nodes’ association to FAPs, and determining the IoT nodes’ transmission power $P_{i,f}$ (effort) and the FAPs’ charging power $P_{f,i}$ (reward) based on the introduced contract-theoretic model.

**Figure 2:**
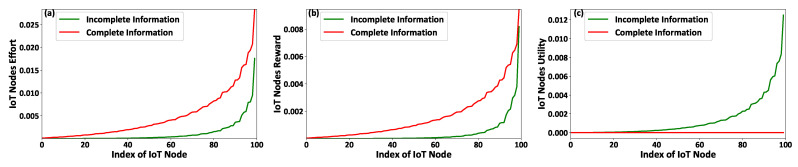
IoT nodes’ (**a**) invested effort, (**b**) gained reward, and (**c**) achieved utility under the proposed contract-theoretic energy harvesting framework—Complete versus Incomplete information scenarios.

Figure 2a–c present the IoT nodes’ effort $q_{i}$, the FAPs’ reward $r_{i}$, and the IoT nodes’ achieved utility $U_{i}$ as a function of the IoT nodes’ types $t_{i}$ considering the scenarios of complete and incomplete information. It is noted that the IoT nodes’ types $t_{i}$ are sorted for presentation purposes, i.e., $t_{1}<t_{2}<\ldots t_{i}<\cdots <t_{\left|I\right|}$. The results reveal that the IoT nodes of higher type, i.e., better socio-physical conditions, invest more effort (Figure 2a) by transmitting with higher transmission power to report more data to the corresponding FAPs that they are associated with. Thus, following the fairness (Proposition 1) and monotonicity (Proposition 2) conditions, the IoT nodes of higher type enjoy a higher reward (Figure 2b) from the FAPs, i.e., higher charging power. Therefore, based on the rationality (Proposition 3) condition, the IoT nodes of higher type achieve a higher utility, as shown in Figure 2c. Furthermore, it should be noted that the FAPs provide the minimum possible rewards to the IoT nodes under the complete information scenario given that they know their socio-physical characteristics; thus, $U_{i}=0,\in I$ (Figure 2c).

**Figure 3:**
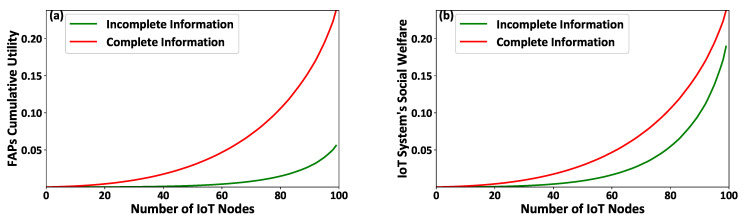
(**a**) FAPs’ cumulative utility and (**b**) the overall IoT system’s social welfare under the proposed contract-theoretic energy harvesting framework—Complete versus Incomplete information scenarios.

Additionally, Figure 3a,b illustrate the FAPs’ cumulative utility and the overall IoT system’s social welfare, respectively. The results show that the overall IoT system operates better under the complete information scenario. Specifically, it is observed that the social welfare of the overall IoT system is reduced, on average, by 67 % under the incomplete information scenario, where the latter is a realistic situation in an IoT system. The latter observation confirms that the proposed smart energy harvesting framework operates in an acceptable manner under the realistic conditions of complete lack of information regarding the IoT nodes’ socio-physical conditions.

### 5.2. Benefits of Socio-Physical Approach

In this section, a detailed comparative analysis is presented in order to highlight the benefits of introducing a contract-theoretic approach to perform the smart energy harvesting and considering the unique socio-physical characteristics of each IoT node. The realistic incomplete information scenario is considered and compared to a type agnostic scenario, where each FAP offers proportional rewards to the IoT nodes based on their invested effort, i.e., $r_{i}\left(q_{i}\right)=\frac{\sum_{i=1}^{\left|I\right|}t_{i}}{\left|I\right|}q_{i}$.

**Figure 4:**
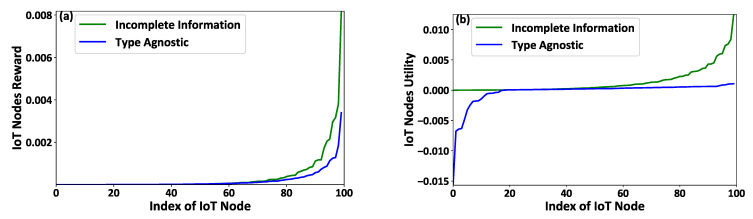
(**a**) IoT nodes reward and (**b**) achieved utility under the contract-theoretic versus the type agnostic framework of energy harvesting.

**Figure 5:**
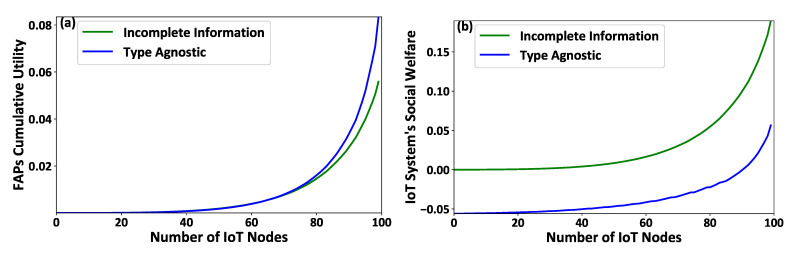
(**a**) FAPs’ cumulative utility and (**b**) the overall IoT system’s social welfare under the contract-theoretic versus the type agnostic framework of energy harvesting.

Figure 4a,b presents the IoT nodes’ received rewards and their corresponding achieved utility, respectively, as a function of the IoT nodes’ IDs. Figure 5a,b depicts the FAPs’ cumulative utility and the overall IoT system’s social welfare, respectively, as a function of the number of IoT nodes in the examined system. The results reveal that the proposed contract-theoretic smart energy-harvesting model exploits the nodes’ socio-physical characteristics in a personalized manner, as compared to the type agnostic model. Thus, the IoT nodes receive rewards tailored to their type (Figure 4a), and the IoT nodes’ that invest a higher effort, given their higher type, receive higher rewards. The achieved benefits are also depicted in the IoT nodes’ achieved utility (Figure 4b), which respects the individual rationality condition under the proposed contract-theoretic model. Thus, the IoT nodes always achieve a positive utility for their invested effort in contrast to the type agnostic scenario. The FAPs’ cumulative utility is similar in both cases (Figure 5a), given that the FAPs gain from under-rewarding some IoT devices, while they spend a great amount of charging power by over-rewarding some other IoT devices in the type agnostic scenario. By studying the overall IoT system (Figure 5b), we observe that the contract-theoretic smart energy harvesting framework outperforms the type agnostic approach by a factor of four on average, given the personalized rewarding mechanism that enables the offering of personalized rewards to the IoT nodes tailored to their needs. Thus, the transmission and charging power usage is intelligently exploited in the system.

### 5.3. Comparative Evaluation

In this section, we demonstrate the benefits of introducing an artificial intelligent method based on reinforcement learning to facilitate the intelligent association of the IoT nodes to the FAPs. Five comparative scenarios are considered in terms of enabling the IoT nodes to select the FAP that they will be associated with: (i) the proposed reinforcement learning mechanism (RL), as introduced in Section 4, the IoT nodes’ select (ii) the closest FAP to connect (Min Distance), (iii) the FAP that offered the maximum charging power in the previous timeslot (Max Charging Power), (iv) the FAP that the minimum number of IoT nodes (Min Nodes) were connected to it in the previous timeslot, and (v) a random FAP (Random). It is noted that all the IoT nodes are within the coverage area of all the considered FAPs. The overall results were derived by performing a detailed Monte Carlo analysis of 1000 executions of the overall framework for all the comparative scenarios.

**Figure 6:**
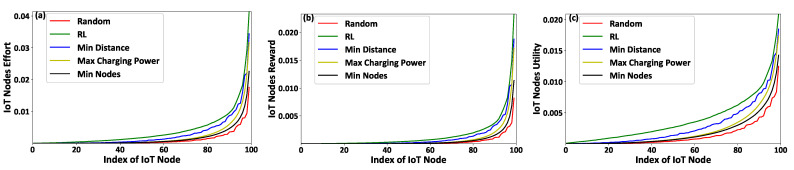
(**a**) IoT nodes’ invested effort, (**b**) gained reward, and (**c**) achieved utility — A Comparative Evaluation.

**Figure 7:**
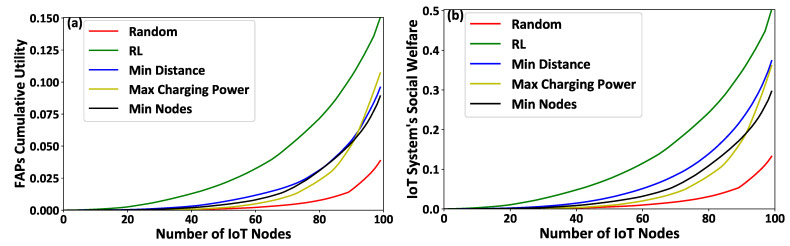
(**a**) FAPs’ cumulative utility and (**b**) the overall IoT system’s social welfare—A Comparative Evaluation.

Figure 6a–c present the IoT nodes’ invested effort, gained reward, and achieved utility, respectively, as a function of the IoT nodes’ IDs. Figure 7a,b illustrate the FAPs’ cumulative utility and the overall IoT system’s social welfare, respectively, as a function of the number of IoT nodes within the overall system. The results show that the proposed framework outperforms compared to all other scenarios, in terms of IoT nodes’ invested effort (Figure 6a), gained reward (Figure 6b), and achieved utility (Figure 6c), FAP’s cumulative utility (Figure 7a), and system’s social welfare (Figure 7b). This observation stems from the proposed reinforcement learning mechanism’s inherent characteristics that enable the IoT nodes to select the FAPs that hollistically provide them with a superior utility in the long term, as compared to considering only fragmented selection criteria, such as the minimum distance, the maximum charging power, and/or the minimum number of connected IoT nodes to the FAPs. It is also observed that FAP selection based on the minimum distance presents the next best results after our proposed reinforcement learning-based framework, as the communication distance is a dominant factor in both the transmission and charging signals’ power attenuation. The random selection scenario presents the worst results, as the IoT nodes make a non-sophisticated selection of FAPs without considering their physical and social characteristics. The Max Charging Power and Min Nodes FAP selection scenarios present similarly mediocre results, as all the IoT nodes tend to select only one FAP per timeslot, and this type of selection creates a burden on the selected FAP to serve all the connected IoT nodes efficiently.

**Figure 8:**
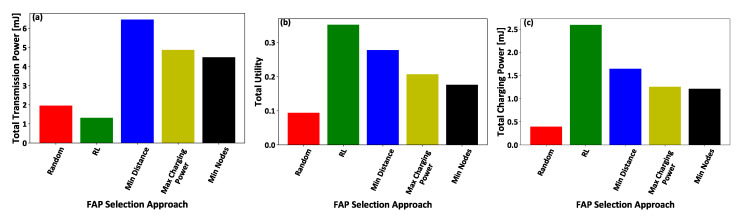
(**a**) Total transmission power and (**b**) utility of all the IoT nodes, and (**c**) total charging power of all the FAPs — A Comparative Evaluation.

Furthermore, Figure 8a–c illustrates the total transmission power and utility of all the IoT nodes, and the total charging power of all the FAPs, respectively, for all the examined comparative scenarios. The results demonstrate that the proposed reinforcement learning-based FAPs’ selection mechanism enables the IoT nodes to transmit with low power (Figure 8a) and efficiently exploit the FAPs’ charging power (Figure 8c), in order to achieve superior utility (Figure 8b) within the examined IoT system. On the other hand, the single selection criterion of FAPs scenarios present worse results, as they provide a myopic view to the IoT nodes regarding the IoT system, and their most beneficial choice of FAP to be connected and transmit information, while also harvesting power. Additionally, the random scenario provides the lowest utility to the IoT nodes, as it is not able to efficiently balance the trade-off between the energy spent to transmit the IoT nodes’ data and the corresponding harvested energy from the FAPs’ radio frequency signals.

**Figure 9:**
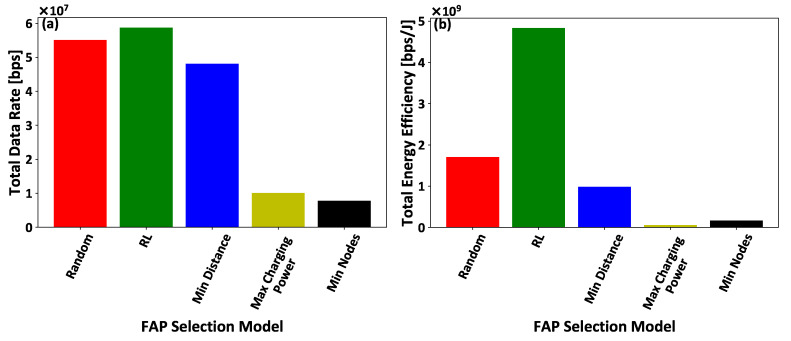
(**a**) Total achieved data rate and (**b**) energy efficiency of all the IoT nodes — A Comparative Evaluation.

Additionally, Figure 9a,b illustrates the IoT nodes’ total achieved data rate and their corresponding total achieved energy efficiency under all the examined comparative scenarios. The results illustrate that the intelligent IoT nodes’ association to the FAPs by exploiting the introduced artificial intelligent framework, results in the better exploitation of the low transmission power (Figure 8a) in order to achieve a superior data rate (Figure 9a) and improved energy efficiency (Figure 9b), compared to the rest of the examined scenarios. It is also illustrated that the comparative scenarios, which perform a myopic selection of FAP for the IoT nodes, achieve low data rate and energy efficiency. Thus, it is concluded that a multi-parameter consideration in the selection of the FAP and providing to the IoT nodes with the intelligence needed to perform the FAP selection, provides better results in terms of the transmission power and achieved data rate, and correspondingly improves the overall energy efficiency of the IoT nodes.

## 6. Conclusions

In this paper, a smart energy harvesting framework for Internet of Things is introduced based on Contract Theory and Reinforcement Learning. Initially, a wireless powered communication system model is introduced, which exploits the IoT nodes’ physical and social characteristics in order to define their types. Then, the IoT nodes’ transmission power and the FAPs’ personalized charging power based on the IoT nodes’ characteristics are determined by introducing a contract-theoretic framework to capture the interactions among the IoT nodes and the FAPs. The scenarios of incomplete and complete information regarding the IoT nodes’ types are examined in detail. Furthermore, an artificial intelligence mechanism is proposed based on reinforcement learning in order to enable the IoT nodes to select the most beneficial choice of FAP to connect to in the long-term. Finally, detailed simulation and comparative results are presented to show the pure operation performance of the proposed framework, as well as its drawbacks and benefits, compared to other approaches.

Our current and future work aims to extend the proposed framework in a 6G operation wireless environment enriched with reconfigurable intelligent surfaces in order to improve the channel conditions among the IoT nodes and the FAPs. To quantifying the benefits introduced by adopting the reconfigurable intelligent surfaces, we perform a detailed experimental analysis to measure the transmission and charging power savings.

## Figures and Tables

**Table 1:** List of Acronyms.

Acronym	Meaning
IoT	Internet of Things
FAP	Femtocell Access Point
QoS	Quality of Service
UAV	Unmanned Aerial Vehicle
WPCN	Wireless Powered Communication Network
WET	Wireless Energy Transfer
WIT	Wireless Information Transmission
NOMA	Non-Orthogonal Multiple Access
SIC	Successive Interference Cancellation
AWGN	Additive White Gaussian Noise
IC	Incentive Compatibility
IR	Individual Rationality

**Table 2:** Summary of Key Notations.

Notation	Description [Units]
*F*	Set of femtocells
*f*	Index of femtocell
*I*	Set of IoT nodes
$d_{i,i^{\prime }}$	Distance among two IoT nodes $i,i^{\prime }\in I$ [m]
$d_{i,f}$	Distance of an IoT node from a FAP
$\tau_{WIT}$	WIT phases’ duration [sec]
$\tau_{WET}$	WET phases’ duration [sec]
τ	timeslot [sec]
$r_{i,i^{\prime }}$	Relationship factor $r_{i,i^{\prime }}\in \left[0,1\right]$ among two IoT nodes
$G_{i,i^{\prime }}$	Channel gain among two IoT nodes $i,i^{\prime }\in I$
$G_{i,f}$	Channel gain among an IoT node and a FAP
$E_{av.i}^{\left(\tau \right)}$	IoT node’s available energy [J]
$P_{i}^{Max(\tau )}$	IoT node’s maximum possible transmission power [W]
$E_{tr.i}^{\left(\tau \right)}$	IoT node’s consumed energy for data transmission [J]
$E_{harv.i}^{\left(\tau \right)}$	IoT node’s harvested energy [J]
$P_{i,f}$	IoT node’s transmission power [W]
$P_{f,i}$	FAP’s charging power for the IoT node *i* [W]
$R_{i,f}$	IoT node’s achievable data rate [bps]
*W*	System’s bandwidth [Hz]
$\sigma^{2}$	Power of zero-mean Additive White Gaussian Noise (AWGN)
$SP_{i}$	Socio-physical factor of the IoT node *i*
$\rho_{i,f}$	Proximity factor of the IoT node *i* to FAP *f*
$\eta_{i}$	Energy conversion efficiency factor of the IoT node *i*
$G_{\Sigma }$	Channel quality vector
${\tilde{G}}_{\Sigma }$	Normalized channel quality vector
$CI_{i,i^{\prime }}$	Communication interest factor
$t_{i},q_{i},r_{i},U_{i}$	IoT node’s type, effort, reward, utility function
*k*	IoT node’s data transmission cost
$e(r_{i}\left(t_{i}\right))$	Evaluation function
*w*	FAP’s cost to provide the rewards
$U_{f}$	FAP’s utility function

## Data Availability

Not Applicable.

## Appendix A. Proofs

### Appendix A.1. Proof of Proposition 1

In the following proof, we examine both the sufficiency, i.e., $t_{i}>t_{i^{\prime }}\Rightarrow r_{i}>r_{i^{\prime }}$, and the necessity, i.e., $r_{i}>r_{i^{\prime }}\Rightarrow t_{i}>t_{i^{\prime }}$ of the condition. Starting with the sufficiency of the fairness condition and based on the Incentive Compatibility (IC) condition Equation (5c), we have $\forall i,i^{\prime }\in I,i\neq i^{\prime }$:

(A1) $$\;t_{i}e\left(r_{i}\right)-kq_{i}\geq t_{i}e\left(r_{i^{\prime }}\right)-kq_{i^{\prime }}\;$$

(A2) $$\;t_{i^{\prime }}e\left(r_{i^{\prime }}\right)-kq_{i^{\prime }}\geq t_{i^{\prime }}e\left(r_{i}\right)-kq_{i}\;$$

By adding Equation (A1) and Equation (A2), we have the following expression:

(A3) $$\;t_{i}e\left(r_{i}\right)+t_{i^{\prime }}e\left(r_{i^{\prime }}\right)\geq t_{i}e\left(r_{i^{\prime }}\right)+t_{i^{\prime }}e\left(r_{i}\right)\;$$

By recognizing the terms in Equation (A3), we have: $\left(t_{i}-t_{i^{\prime }}\right)e\left(r_{i}\right)\geq \left(t_{i}-t_{i^{\prime }}\right)e\left(r_{i^{\prime }}\right)$, and given that $t_{i}>t_{i^{\prime }}$, we conclude that $e\left(r_{i}\right)>e\left(r_{i^{\prime }}\right)$. Given that the evaluation function $e(r_{i})$ is strictly increasing with respect to the reward $r_{i}$, we conclude that $r_{i}>r_{i^{\prime }}$. Thus, we have shown so far that $t_{i}>t_{i^{\prime }}\Rightarrow r_{i}>r_{i^{\prime }}$ holds true.

Continuing with the necessity condition, we know that $r_{i}>r_{i^{\prime }}$ and the evaluation function is strictly increasing; thus, $e\left(r_{i}\right)>e\left(r_{i^{\prime }}\right)\Leftrightarrow e\left(r_{i}\right)-e\left(r_{i^{\prime }}\right)>0$. Based on Equation (A3), we have: $t_{i}\left(e\left(r_{i}\right)-e\left(r_{i^{\prime }}\right)\right)\geq t_{i^{\prime }}\left(e\left(r_{i}\right)-e\left(r_{i^{\prime }}\right)\right)$; thus, we conclude that $t_{i}>t_{i^{\prime }}$. Therefore, we have shown that $r_{i}>r_{i^{\prime }}\Rightarrow t_{i}>t_{i^{\prime }}$ holds true. Finally, it is noted that $r_{i}=r_{i^{\prime }}\Leftrightarrow t_{i}=t_{i^{\prime }}$, by easily following the above reasoning.

### Appendix A.2. Proof of Proposition 2

Based on Proposition 1, we have $r_{1}<\cdots <r_{i}<\cdots <r_{\left|I\right|}\Leftrightarrow t_{1}<\cdots <t_{i}<\cdots <t_{\left|I\right|}$. The reward is defined as a strictly increasing function of the IoT node’s invested effort. The physical meaning of this definition is that an IoT node which spends more energy to transmit its data to the FAP, should be charged more by the FAP in order to remain active in the IoT network. Thus, we can easily conclude with the outcome $t_{1}<\cdots <t_{i}<\cdots <t_{\left|I\right|}\Leftrightarrow r_{1}<\cdots <r_{i}<\cdots <r_{\left|I\right|}\Leftrightarrow q_{1}<\cdots <q_{i}<\cdots <q_{\left|I\right|}$.

### Appendix A.3. Proof of Proposition 3

For two representative IoT nodes, $i,i^{\prime },\forall i,i^{\prime }\in I,i\neq i^{\prime }$, we write their incentive compatibility conditions as follows: $t_{i}\cdot e\left(r_{i}\right)-kq_{i}\geq t_{i}e\left(r_{i^{\prime }}\right)-kq_{i^{\prime }}\overset{t_{i}>t_{i^{\prime }}}{\Leftrightarrow }t_{e}\left(r_{i}\right)-kq_{i}>t_{i^{\prime }}e\left(r_{i^{\prime }}\right)-kq_{i^{\prime }}$. Thus, we concluded that $U_{i}>U_{i^{\prime }}$. By generalizing this analysis, we conclude that $t_{1}<\cdots <t_{i}<\cdots <t_{\left|I\right|}\Leftrightarrow U_{1}<\cdots <U_{i}<\cdots <U_{\left|I\right|}$.

### Appendix A.4. Proof of Theorem 1

Based on the individual rationality constraint Equation (6b), we consider the minimum achieved utility that is acceptable by the IoT node, i.e., $t_{i}e\left(r_{i}\right)-kq_{i}=0$, in order to participate in the IoT network. Thus, for $e\left(r_{i}\right)=\sqrt{r_{i}}$, we have $r_{i}={\left(\frac{kq_{i}}{t_{i}}\right)}^{2}$. Based on Equation (6a), by taking the first-order derivative with respect to $q_{i}$ equal to zero, we have $q_{i}^{*}=\frac{t_{i}^{2}}{2wk^{2}}$, thus, $r_{i}^{*}={\left(\frac{t_{i}}{2wk}\right)}^{2}$.

### Appendix A.5. Proof of Lemma 1

We consider three indicative IoT nodes: i−1,i,i+1, and we write the IC conditions, as follows

(A4) $$t_{i+1}e\left(q_{i+1}\right)-kq_{i+1}\geq t_{i+1}e\left(q_{i}\right)-kq_{i}$$

(A5) $$t_{i}e\left(q_{i}\right)-kq_{i}\geq t_{i}e\left(q_{i-1}\right)-kq_{i-1}$$

The evaluation function is strictly increasing; thus, we have: $e\left(q_{i}\right)-e\left(q_{i-1}\right)\geq 0$. We also have: $t_{i+1}>t_{i}\Leftrightarrow t_{i+1}\left(e\left(q_{i}\right)-e\left(q_{i-1}\right)\right)\geq t_{i}\left(e\left(q_{i}\right)-e\left(q_{i-1}\right)\right)\geq^{Equation\;(A5)}k\left[q_{i}-q_{i-1}\right]$. We recursively perform the latter analysis and we have: $t_{i+1}e\left(q_{i+1}\right)-kq_{i+1}\geq t_{i+1}e\left(q_{i-1}\right)-kq_{i-1}\geq t_{i+1}e\left(q_{i-2}\right)-kq_{i-2}\geq \cdots \geq t_{i+1}e\left(q_{1}\right)-kq_{1}$. Thus, we conclude with the equivalent local downward IC constraint.

(A6) $$t_{i}e\left(q_{i}\right)-kq_{i}\geq t_{i}e\left(q_{i-1}\right)-kq_{i-1}$$

### Appendix A.6. Proof of Lemma 2

We consider three indicative IoT nodes: i−1,i,i+1, and we write the following IC constraints:

(A7) $$t_{i-1}e\left(q_{i-1}\right)-kq_{i-1}\geq t_{i-1}e\left(q_{i}\right)-kq_{i}$$

(A8) $$t_{i}e\left(q_{i}\right)-kq_{i}\geq t_{i}e\left(q_{i+1}\right)-kq_{i+1}$$

Based on Equation (A8) and the fairness condition, we have:

(A9) $$k\left(q_{i+1}-q_{i}\right)\geq t_{i}\left[e\left(q_{i+1}\right)-e\left(q_{i}\right)\right]\geq^{t_{i}>t_{i-1}}t_{i-1}\left[e\left(q_{i+1}\right)-e\left(q_{i}\right)\right]$$

Based on Equations (A7) and (A9), we write: $t_{i-1}e\left(q_{i-1}\right)-kq_{i-1}\geq t_{i-1}e\left(q_{i}\right)-kq_{i}\geq t_{i-1}e\left(q_{i+1}\right)-kq_{i+1}$. Thus, we have $t_{i-1}e\left(q_{i-1}\right)-kq_{i-1}\geq t_{i-1}e\left(q_{i+1}\right)-kq_{i+1}$, which shows that if the local downward IC constraint holds true, then all the upward constraints also hold true, and we have: $t_{i-1}e\left(q_{i-1}\right)-kq_{i-1}\geq t_{i-1}e\left(q_{i+1}\right)-kq_{i+1}\geq \cdots \geq t_{i-1}e\left(q_{\left|I\right|}\right)-kq_{\left|I\right|}$.
